# From Field Data to Practical Knowledge: Investigating the Bioecology of the Oak Lace Bug—An Invasive Insect Species in Europe

**DOI:** 10.3390/insects14110882

**Published:** 2023-11-15

**Authors:** Flavius Bălăcenoiu, Dragoș Toma, Constantin Nețoiu

**Affiliations:** 1 National Institute for Research and Development in Forestry “Marin Dracea”, Eroilor 128, 077190 Voluntari, Romania; dragost93@gmail.com (D.T.); c_netoiu@yahoo.com (C.N.); 2 Faculty of Silviculture and Forest Engineering, Transilvania University of Brașov, Sirul Beethoven 1, 500123 Brașov, Romania

**Keywords:** biological invasions, insect pests, development model, degree days, forest management

## Abstract

**Simple Summary:**

In a world filled with diverse ecosystems, understanding the behaviour of invasive species is crucial for maintaining balance and health. This study delves into the bioecology of the invasive oak lace bug in Europe, shedding light on its life cycle through a degree day-based model and presenting insights gathered from field-based life tables. Our research addresses key knowledge gaps, offering valuable information for effective pest control in forest ecosystems. By bridging the gap between scientific exploration and practical implications, we aim to empower the public with accessible knowledge on how to protect European forests and maintain the delicate equilibrium of natural environments.

**Abstract:**

*Corythucha arcuata*, commonly known as the oak lace bug (OLB), is an insect species originally native to North America that has become an invasive species of significant concern in Europe. This invasive pest has been observed in various European countries, raising concerns about its impact on forest ecosystems. In 2015, it was first documented in Romania, further highlighting the need for research on its bioecology and life cycle. This study investigated the bioecology of the OLB in the southern region of Romania, focusing on its life cycle, development, and population dynamics. The results indicated that the OLB has three generations per year and overwinters in the adult stage in sheltered locations. Temperature significantly influenced the timing of egg hatching, nymph appearance, and adult development, with variation observed between generations. Additionally, a life table analysis provided insights into the population dynamics of the OLB in its natural environment, revealing variation in egg laying trends across generations. This research contributes to a better understanding of the OLB’s bioecology and provides essential data for forest managers developing science-based management strategies to mitigate its impact. By elucidating the life cycle and development patterns of the OLB in southern Romania, this study aids in the development of predictive models and life tables tailored to the region. These findings empower forest managers with the knowledge needed to make informed decisions for effective OLB management, ultimately preserving the health of forest ecosystems.

## 1. Introduction

A species that is introduced to a new territory by human activities, whether deliberately or unintentionally, is considered an alien species [1,2,3,4]. If such a species manages to reproduce, spread rapidly (at considerable distances from the introduction site), and have a negative impact on the newly occupied territory, it becomes an invasive alien species, and the phenomenon is defined as a biological invasion [1,2,3,4,5,6,7,8]. These invasive alien species can cause a wide range of negative effects on the environment, economy, and human health [5,8,9,10,11,12,13,14,15,16].

Furthermore, invasive alien species are considered a major factor in the extinction of certain species, posing a significant threat to biodiversity [17,18]. Kenis & Branco [19] argue that, although European forest ecosystems are less affected by invasive species than forests in other continents, a wide variety of potentially harmful invasive species has recently become established in European forests. They have a substantial impact on society and the bioeconomy, posing challenges to sustainable forest management and necessitating effective management strategies.

*Corythucha arcuata* (Say, 1832) (Hemiptera, Tingidae), commonly known as the oak lace bug (OLB), is an insect of North American origin that is considered an invasive alien species in Europe. Its natural range extends across the southern part of Canada and several states in the eastern USA [20,21]. According to studies conducted in the Nearctic region of North America [20,21], it develops on host plants such as *Quercus alba* L., *Quercus macrocarpa* Michaux, *Quercus montana* Willdenow, *Quercus muehlenbergii* Engelmann, *Quercus prinoides* Willdenow, *Quercus rubra* L., and other species of *Quercus* (Fagaceae). Additionally, the insect has occasionally been observed on host species from the genera *Acer*, *Malus*, and *Rosa*.

In Europe, the OLB was first observed in northern Italy in 2000 [22]. Two years later, in 2002, it was documented in southern Switzerland in flight interception traps set between April and July [23]. In the same year (2002), the OLB was also detected in Turkey, approximately 200 km east of Istanbul [24]. In 2005, a specimen of this species was identified in Iran [25]. For several years, the OLB was not reported in new countries, but in 2012, it was observed for the first time in Bulgaria [26], followed by sightings in Hungary [27], Croatia [28], and Serbia [29,30] in 2013. In 2015, the insect was first observed in Russia, in Krasnodar [31]. Additionally, in August 2015, it was reported for the first time in Romania [32,33]. In the following year, 2016, *Corythucha arcuata* was observed for the first time in several countries, including Albania [34] and Slovenia [35]. In 2017, it appeared in three new countries: Bosnia and Herzegovina [36], France [37], and Ukraine [34,38]. In May 2018, the OLB was first reported in northeastern Greece [34], and, in June 2018, in southern Slovakia [39]. A study in 2019 confirmed the presence of the insect in 21 locations in Austria [40].

In the context of biological invasions caused by insects in forest ecosystems, understanding pest behaviour can contribute to identifying favourable conditions for outbreak initiation, enabling forest managers to make science-based decisions regarding species control. To manage a pest effectively and scientifically, it is necessary to identify the feedback processes that regulate its population [41,42]. In the implementation of integrated pest management, obtaining information regarding pest population abundance and the environmental conditions in which a pest thrives is essential, as is understanding the factors that influence population development [43,44].

Based on the aforementioned considerations, we believe that in-depth research on the bioecology of the OLB is necessary. Hence, our primary objective was to contribute to the advancement of the scientific understanding of the bioecology of this invasive forest insect species. The expected outcomes of this work included obtaining a definitive description of the insect’s life cycle, the generation of an insect development prediction model based on degree days, and the development of life tables in the natural environment of the OLB. Using our new insights into the bioecology of the OLB, forest managers will be able to make informed decisions regarding the management of forests and this invasive species based on Romania-specific scientific foundations.

## 2. Materials and Methods

### 2.1. Study Area and Data Collection

To achieve our objectives, an intensive monitoring approach was employed to track the hatching and development of individuals, transitions between stages, and temporal variation in the number of individuals at each stage across all three generations. Consequently, during the 2020–2021 vegetation seasons, we set up an experiment based on controlled growth of *C. arcuata*, spanning from egg to adult, covering the progression from the first generation to the third generation at the conclusion of the cycle.

**Figure 1:**
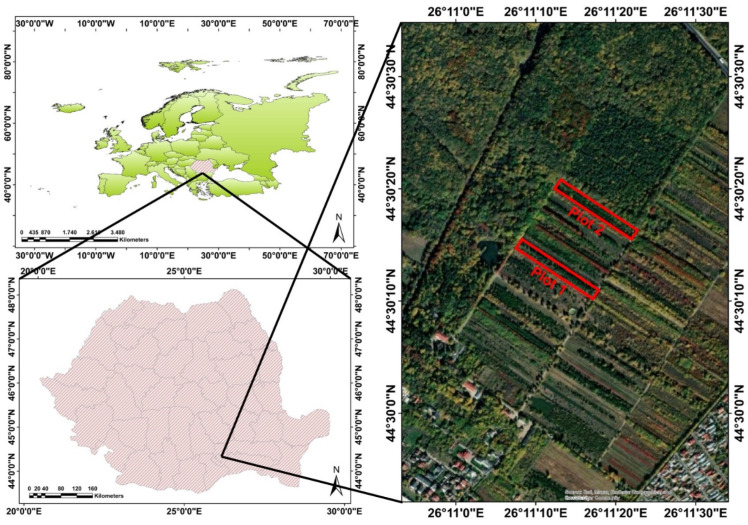
Maps of the locations of the study and experimental plots (Esri, Maxar, Earthstar Geographics, and the GIS User Community).

**Figure 2:**
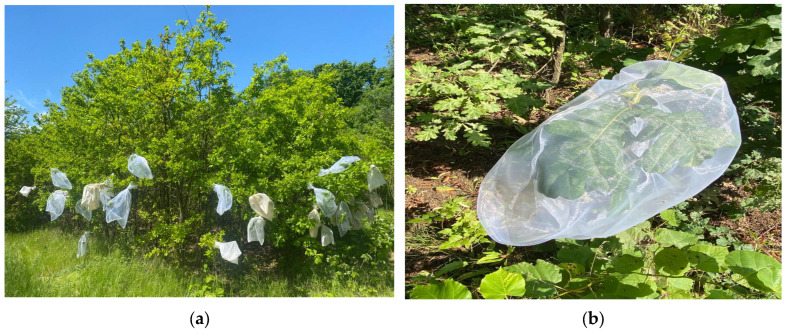
Experimental growth environment and insect isolation: (**a**) Controlled growth environment in experimental plots; (**b**) cloth bags designed for controlled growth of insects.

Although the experiment was centred around the controlled growth of insects, to ensure the monitored individuals could develop under favourable conditions, they were reared in a natural setting. To fulfil this objective, within the National Institute for Research and Development in Forestry “Marin Drăcea” Nursery (NIRDF), two identical plots were selected (Figure 1). In Plot 1, insects from the first and third generations were reared, while in Plot 2, insects from the second generation were reared. These plots were positioned approximately 50 metres apart and exclusively featured common oak saplings (approximately 10 years old), which greatly facilitated the monitoring process (Figure 2a).

To isolate the insects, cloth bags designed for controlled growth were crafted, allowing individuals to develop under favourable conditions. Each bag contained a pair comprising one male and one female, and the bag was subsequently sealed and attached to a tree (Figure 2b). Following the numbering and marking of the bags, they were monitored every 1–3 days; we documented any changes relative to the previous observation.

For each generation, a minimum of 35 bags (70 male–female pairs) were installed, with their placement occurring at different times depending on the generation (Table 1).

To monitor the development of Generation I, pairs of adults from the overwintering generation were selected. These adults were isolated after they emerged from hibernation, immediately upon becoming active, in the plot designated for Generation I. Subsequently, each bag was intensively monitored until the emergence of the adults. Among the adults resulting from the first generation, additional pairs were selected to develop Generation II. In early July, these pairs were transferred and isolated in the plot designated for Generation II. As with the previous generation, each bag was intensively monitored until the individuals of Generation II reached adulthood. Finally, among the adults resulting from the first generation, additional pairs were selected, and in early August, they were transferred and isolated in the plot designated for Generation III. The process continued as with the previous generations, with each pair being intensively monitored until the individuals of Generation III reached adulthood. The selection of male–female pairs from one generation to the next was conducted to avoid inbreeding.

Climatic data were obtained from the meteorological station at the NIRDF, which is located approximately 100 metres from the plots where the monitoring was conducted. Initially, we collected a comprehensive set of climatic data, including minimum and maximum temperatures, as well as average values. Subsequently, we focused on specific factors relevant to our research objectives.

### 2.2. Developing the Insect Development Prediction Model Based on Degree days

The degree day calculation was conducted using the mean method (1) [45,46,47,48], which integrates both the daily minimum and maximum temperatures, along with the base temperature.

(1) $$DD=\left(\frac{Tmax+Tmin}{2}\right)-Tb$$

where:

DD = accumulated degree days for a day;

Tmax = maximum temperature of the respective day;

Tmin = minimum temperature of the respective day;

Tb = base temperature specific to the pest.

Insect development occurs within the climatic range between the lower and upper temperature thresholds (characteristic each species) and halts when the temperature drops below this lower limit. In the calculation of degree days, the temperature corresponding to the lower threshold is used as the “base temperature”. Considering that a specific base temperature for the pest *C. arcuata* was not identified upon reviewing the specialized literature, a value of 50 °F, equivalent to 10 °C, was adopted. This value is recommended for species for which the lower threshold has not been determined [48].

Therefore, if the daily average temperature is one degree above the base temperature of 10 °C, it means that one degree day has accumulated on that day. Negative results (when the daily average temperature is below 10 °C) are disregarded. At the beginning of the season, degree days accumulate slowly, but as the daily average temperature starts to rise, the degree day value increases exponentially [48]. Data for calculating degree days were collected starting from 1 January.

For example, in the conditions of the year 2020, until 2 February, no daily average temperature exceeded the threshold of 10 °C. On 2 February, the daily average temperature was 11.78 °C, and on 3 February, it was 11.95 °C. Applying the mean method (1), starting from 1 January 2020 to 3 February, 3.73 degree days were accumulated.

Using the mean method (1), during the period from 2020 to 2021, the number of degree days accumulated until the appearance of each developmental stage of each monitored pair was observed for all three developed generations. Thus, by considering all the resulting data, it was possible to create a predictive model for insect development based on degree days.

### 2.3. Constructing the Life Table of the Insect Population in a Natural Environment

We employed the “age-specific” life table method to construct the life table, also known as the “horizontal table”. This method involves tracking the development of a real, controlled cohort of individuals within the population, all belonging to the same generation and age group, without overlapping with other generations [49]. Given that many insects exhibit discrete generations and non-stationary populations (as is the case with OLB), the age-specific life table is more widely applicable than the time-specific life table [50].

For each of the three generations, the construction of the life table started with the total number of pairs established and introduced into the experiment (*N*′). Additionally, in designing the life table, only the number of successful pairs was considered. The number of successful pairs (*N*) is represented by the number of pairs that successfully completed the development cycle of the respective generation. For example, if one of the pairs encountered developmental issues in the later nymph stages or even in the adult stage due to abiotic factors, it was excluded from the final calculation so as not to negatively influence the construction of the life table.

Furthermore, in designing the life table, for each developmental stage and generation, the completion criteria developed by Harcourt [51] were considered. The aim was to determine the average number of eggs per pair (*lx_eggs_*), the mortality and infertility of the eggs (*dx_eggs_*), the average number of nymphs (*lx_nymphs_*), the mortality of nymphs (*dx_nymphs_*), the average number of adults (*lx_adults_*), the sex ratio (*lx_female_*), and the generation survival (GS). However, it is important to emphasize that we adapted this methodology to align it with the specific developmental cycle of OLB, as outlined in Table A1. By doing so, we aimed to tailor the methodology to the unique characteristics of this insect species, ensuring that the life table reflects the intricacies of its life stages and development.

To determine the gender and age of OLB individuals, we relied on the well-documented morphology studied and described extensively, both in its native habitat of North America since the early 20th century [21,52] and in regions recently invaded in Europe and Asia [22,53].

### 2.4. Data Analyses

As an initial step, the normality of the distribution of the count data was verified using Shapiro–Wilk tests, while homogeneity of variance was assessed using Levene’s test. Given that the results of these tests confirmed the assumptions of a normal distribution and homogeneity of variance, the data met the requirements for parametric tests. We proceeded to the second step, in which a two-way analysis of variance (ANOVA) was applied.

Data analysis was performed using Statistica 8.0 software (StatSoft Inc, 2007).

## 3. Results

### 3.1. Life Cycle and Number and Durations of Generations

**Figure 3:**
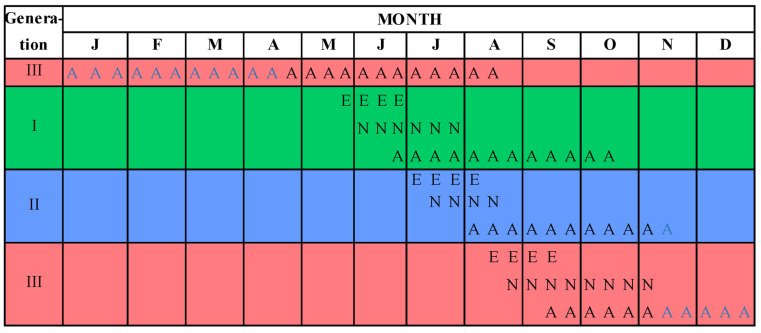
Insect phenology in the environmental context of southern Romania. A = overwintering adults; A = active adults; E = eggs; N = nymphs. Green = the first generation; blue = the second generation; red = the third generation.

According to the observations conducted in the climatic conditions of southern Romania, the OLB had three generations per year and overwintered in the adult stage in sheltered locations. The observations made on the insect in the controlled growth experiment in a natural environment allowed for the construction of a phenogram that represents the life cycle of the OLB in the forests of southern Romania (Figure 3).

Overwintered adults, originating from the previous year, became active in the second week of April, when they emerged from sheltered locations (such as cracks in bark) and flew to reach the newly developed leaves of host trees. They fed until the end of May, when females laid their first eggs. Females continued to lay eggs in multiple batches until the end of June, when the first nymphs of the first generation, originating from the first-laid eggs, appeared at the beginning of June. Although the nymphs developed very quickly, they continued to emerge until the end of July, hatching from eggs laid later. In late June, the first adults of the first generation had already appeared, and this emergence process continued until mid-July. Adults of the first generation could survive until October.

The second generation began in early July, with the first eggs being laid by adults of the first generation. Females continued to lay eggs until the beginning of August. The first nymphs of the second generation started emerging at the end of July and continued to appear until the beginning of August. In the first part of August, adults of the second generation appeared; this process continued until the end of August. Our observations showed that adults of the second generation could survive until the end of the growing season, with the hardiest individuals overwintering along with those of the third generation.

The third generation began in the second half of August, when the first eggs were laid by adults of the second generation. Egg laying continued until the middle of September. Nymphs appeared at the end of August, coinciding with the hatching of the first eggs, and continued to emerge until second half of September. It is important to note that not all nymphs developed into adults, and some persisted until early November. Adults of the third generation appeared in the second half of September and were active for a month, after which they withdrew for overwintering before resuming the development cycle described above in the following year.

### 3.2. Using Degree Days to Predict Insect Development

**Figure 4:**
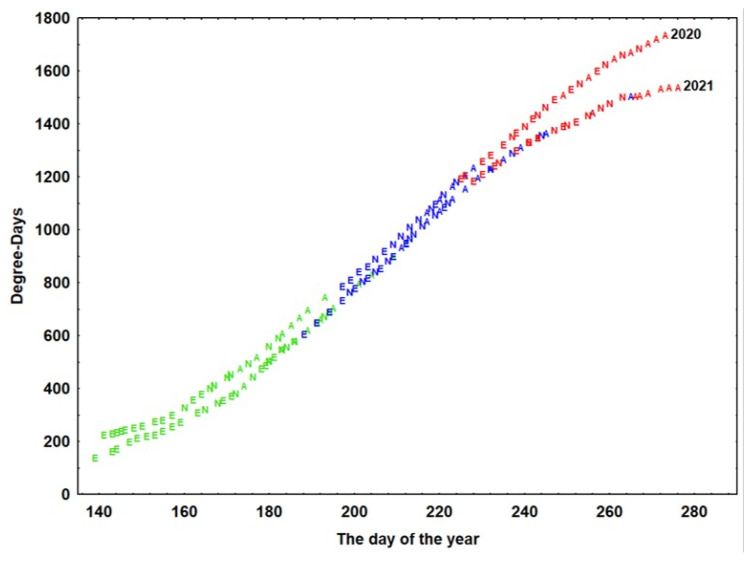
Insect development prediction model based on degree days. A = adults; E = eggs; N = nymphs. Green = the first generation; blue = the second generation; red = the third generation.

According to the model developed, the hatching of eggs, appearance of nymphs, and development of adults were influenced by temperature and showed significant variation between generations (Figure 4).

For the appearance of eggs giving rise to the first generation, a minimum of 135 degree days was recorded, but eggs continued to appear for up to 517 degree days (both values recorded in 2021). Until the appearance of the first nymphs, a minimum of 317 and a maximum of 762 degree days were recorded (both in 2021). Adults of the first generation required a minimum of 408 degree days, but they continued to appear until nearly 900 degree days (observations from 2021).

For the appearance of eggs of the second generation, a minimum of 604 degree days was necessary, but eggs continued to appear for up to almost 1100 degree days. Nymphs appeared from 762 degree days to 1354 degree days (both values recorded in 2021). The first adults of the second generation appeared starting after the accumulation of 931 degree days and continued to appear until 1500 degree days (according to data from 2021).

For the appearance of eggs giving rise to the third generation, 1180 degree days were required (recorded in 2021), but eggs continued to appear for up to nearly 1600 degree days (recorded in 2020). Nymphs needed a minimum of 1190 degree days but continued to appear for up to almost 1700 degree days (observations from 2020). The first adults of the third generation appeared after the accumulation of 1346 degree days (recorded in 2021), and the last adults appeared after 1733 degree days (according to data from 2020).

### 3.3. Life Table of the Insect Population in a Natural Environment

**Figure 5:**
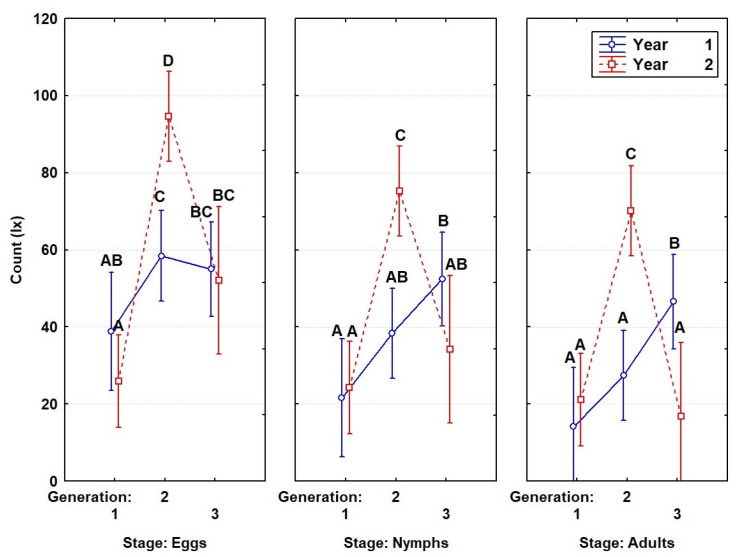
Trends in the insect population across generations. The differences between the means (marked with letters A, B, C, and D) were statistically significant (*p* < 0.05), according to a two-way ANOVA.

Our statistical analysis of the data from the 2020 observations, as presented in Figure 5 and Table 2, showed that adults from the overwintered generation gave birth to the first generation, laying an average of 38.9 eggs per pair. The success rate of the first egg laying was 56%, resulting in an average of 21.6 first-instar nymphs per pair from each egg laying. After progressing through the five nymphal stages, an average of 14.1 adults emerged, with 40% of them being females. In 2021, adults from the overwintered generation laid an average of 25.9 eggs per pair, which gave rise to 24.3 nymphs on average (a success rate of 94%), resulting in 21.1 adults, with half of them being females.

After isolating other pairs obtained from the first generation in 2020, an average of 58.4 eggs per pair was found; this number was significantly higher than the number of eggs from the previous generation in the same year (*p* = 0.047). The success rate of egg laying was 66%, resulting in an average of 38.4 first-instar nymphs per pair from a single egg laying. After passing through the five nymphal stages, the nymphs produced an average of 27.4 adults per pair, with 50% of them being females; this number was significantly higher than in the previous generation from the same year (*p* = 0.049). In 2021, for the development of a new generation, the first generation laid an average of 94.6 eggs; this was significantly higher than in the previous generation (*p* < 0.001). Furthermore, an average of 75.3 nymphs was found, from which an average of 70.1 adults developed, with half of them being females; this number was significantly higher than in the previous generation (*p* < 0.001).

To continue the observations of the development of the third generation, other pairs of adults obtained from the second generation were isolated and grown under controlled conditions in a natural environment. In 2020, we recorded an average of 55 eggs per pair, significantly more than those laid by the overwintered generation (*p* = 0.048) but not significantly different from those laid by the second generation (*p* > 0.05). From the eggs laid, an average of 52.4 nymphs emerged, resulting in a hatching success rate of 95%. Finally, after progressing through the five nymphal stages, the nymphs produced an average of 46.5 adults; this number was significantly higher than in the previous generations (first generation: *p* < 0.001; second generation: *p* = 0.001). Like in the previous generation, females accounted for 50% of the adult population. In 2021, we recorded an average of 52.1 eggs per pair, significantly less than the number of eggs laid by the previous generation (*p* = 0.004) but not significantly more than those laid by the overwintered generation (*p* > 0.005). These pairs produced 34.2 nymphs and, ultimately, 16.9 adults, with half of them being females. The number of adults in the third generation was significantly lower than in the second generation (*p* < 0.001) but not significantly lower than in the first generation (*p* > 0.005).

### 3.4. Analysis of Differences in Egg Laying Trends among Generations

Females’ fertility varied among generations. A female could lay her eggs in 1–9 clusters on the undersides of leaves. The number of eggs in a cluster ranged from 1 or 2 to a maximum of 125. Average values and other statistical parameters for clusters per female and eggs per cluster per generation are presented in Table 3.

According to our data analysis, in 2020, the average number of clusters per female significantly increased from one generation to the next (*p* < 0.001 for Generation I compared to Generation II; *p* = 0.041 for Generation II compared to Generation III). In 2021, the significant increasing trend in the number of clusters persisted between the first and second generations (*p* < 0.001), but for the third generation, the change was no longer statistically significant (*p* > 0.05).

We observed different dynamics in 2020 and 2021 for the average number of eggs per cluster of different generations. In 2020, Generation I laid significantly fewer eggs per cluster compared to Generation II (*p* = 0.002). Between Generation II and Generation III, we did not observe a significant difference (*p* > 0.05). In 2021, we noted that the number of eggs per cluster significantly increased between Generation I and Generation II (*p* < 0.001). Then, we observed a significant decrease between Generation II and Generation III (*p* = 0.006).

## 4. Discussion

This study aimed to understand the life cycle and adaptive responses of the OLB in the context of its invasion in Europe, with a focus on Romania. The main objective of this study was to make a significant contribution to the understanding of the OLB’s biology and ecology in a new environment and emphasize the importance of this knowledge for the sustainable management of oak forest ecosystems.

### 4.1. Life Cycle and Number and Durations of Generations

Based on our observations, the OLB has three generations per year, with hibernation of the adult stage in sheltered locations. Adults have a high potential to withstand short-term exposure to low temperatures [54].

The results of studies from Italy [55] and Hungary [56] confirm that the findings presented in this paper are consistent with the biology of the insect in the invaded area of Europe. There are many similarities between our data and those from previous studies, suggesting uniformity in the development of this species in various geographical regions. However, in the native range of this pest in the state of Delaware (USA), the insect has two complete generations per year plus a third partial generation [20]. One explanation for this difference could be the milder and more temperate climate in Europe, which can provide favourable conditions for the development of three complete generations of the insect. In contrast, in its native area in the USA, more pronounced climatic variation and different environmental conditions could limit the complete development of generations, leading to shorter and incomplete life cycles. Additionally, pressures from natural enemies in the USA, such as parasitic wasps, predatory assassin bugs, lacewing larvae, lady beetles, jumping spiders, pirate bugs, and mites [57], may play a role in determining the number of complete or incomplete generations. This difference in life cycles could represent an adaptation of the species to the specific environment and climatic conditions of each region.

### 4.2. Using Degree Days to Predict Insect Development

The data discussed in the previous subsection originate from observations in Romania, with comparisons to research conducted in the USA [20,21], Northern Italy [55], and Hungary [56]. These comparisons reveal that climatic and biological factors likely shape the species’ life cycle. Insects are poikilothermic, meaning their body temperature is influenced by ambient temperature, and meteorological factors significantly impact their biology, including their metabolic activity, abundance, and dispersal [58,59]. Among the myriad environmental factors, air temperature holds a paramount status in influencing insect behaviour [60]. It plays a pivotal role in metabolic processes, metamorphosis, mobility, and host accessibility, which consequently can lead to fluctuations in their population and dynamics [61]. These notations were not far-fetched; even in the case of the OLB, its diurnal and seasonal dynamics are directly influenced by temperature [62].

These findings suggest that degree day-based development is an efficient method for monitoring and managing insect populations, regardless of regional or climatic variation. The use of calendar-based data to predict the biological cycle of insect pests is considered rudimentary and inefficient [48]. Given that insects are ectothermic, with their body temperatures and development affected by external temperature, a phenological model based on degree days is considered much more suitable for integrated pest management than one based on calendar days [46]. According to the degree day model, each insect species needs to accumulate a certain amount of heat to progress through its life stages, such as egg laying and hatching or adult flight. When considering specific activities related to insect control, such as detection or control at a certain stage of development, the degree day prediction model is much more efficient than the calendar-based model.

Our findings regarding the OLB’s degree day-based life cycle offer a detailed view of this complex process. Data collected directly in natural environments have provided insight into critical moments in the species’ development across its generations. Field-based degree day models are crucial because laboratory-based models are rarely accurate predictors of insect development under natural conditions [63,64]. Through comparing our data with studies conducted in the native habitat of the *Corythucha* spp. in the United States, we observed significant differences in temperature requirements for insect development. In the U.S., lace bugs developed two generations per year, requiring between 239–363 degree days and 1266–1544 degree days, respectively, at a base temperature of 50 °F, calculated from 1 March [65,66,67]. In contrast, in southeastern Romania, we observed that the OLB undergoes three generations per year and exhibits different temperature requirements for development. While these results contribute to our understanding of regional or interannual variation in degree day-based development, the lack of detailed data on the OLB’s degree day requirements in Europe limits direct comparisons. The model above cannot replace field observations but can aid in predicting phenological events. Parallel field observations could help to confirm the reliability of the prediction model, especially for the OLB, an invasive species that can alter its lifestyle across years.

### 4.3. Life Table of the Insect Population in a Natural Environment

Life tables are often used by ecologists to monitor changes occurring in various stages of a studied population. In entomology, they are considered an important tool for understanding changes in the populations of pest insects throughout different stages of their development [49,50,51].

Considering previous research, such as the study conducted by Hosseini-Tabesh et al. [68], which highlighted significant differences in the survival rates, fecundity, and longevity of insects based on their rearing environment (laboratory vs. field), and considering that the OLB is an invasive insect that can exhibit different behaviours in natural environments than under laboratory conditions, we chose to collect field data to obtain a more realistic and applicable understanding of the biology and ecology of this species.

Based on the data from the life table developed for the OLB, differences in the population dynamics of the insect between the first and second years of the study were notable. In the first year, there was a clear increase in vitality and prolificacy as the generations progressed, whereas in the second year of research, variation in vitality and prolificacy was observed between generations (with an increase from the first to the second generation, followed by a decrease from the second to the third generation). Both the increase in the first year and the variation in the second year could result from more complex and unpredictable environmental factors, such as optimal climatic conditions/climatic fluctuations, sufficient/insufficient food resources, and interactions with other biotic factors. Factors like high and low temperature thresholds, fluctuating humidity, or wavelength can affect ovulation, fecundity rate, development, survival, or reproduction [69].

To accurately assess the influence of these factors, it is essential to compare our results with that of previous studies that have investigated insect development at constant temperatures. However, to date, we have not identified any such studies for the OLB. Nonetheless, the results of a study that developed a life table for *Corythucha ciliata* (Say, 1832) (Heteroptera, Tingidae) at five constant temperatures indicate that, as the air temperature increased from 19 °C to 30 °C, both the average fecundity and rate of successfully completing all developmental stages significantly increased [70].

Interestingly, the sex ratio remained approximately 1:1 in all three generations in both years of the study. This constancy may suggest that OLB populations have developed adaptations to maintain this sexual balance. However, the sex ratio is often affected by both intrinsic and extrinsic factors, including genetics, behaviour, physiology, intracellular endosymbionts, and biotic and abiotic factors like temperature, photoperiod, humidity, light conditions, and host or prey quality and quantity [71,72]. A study on the sex ratio of *Nysius huttoni* (White, 1878) (Hemiptera, Lygaeidae), conducted both in the laboratory and using samples from the natural environment [73], revealed that, in the natural environment, the sex ratio remained constant at around 1:1. In contrast, in the laboratory environment, a combination of short photoperiods and low temperatures produced the highest proportion of males. Additionally, Ju et al. [68] showed that the sex ratio of *C. ciliata*, a species similar to the one in this study, could be influenced by air temperature.

Significant variation in the survival of generations (the proportion of individuals that successfully reached the adult stage) was observed between generations and between study years. In the first year, there was a clear increasing trend from the first to the third generation, suggesting positive adaptation to environmental conditions. In contrast, in the second year, we witnessed a reversal of this trend. The causes of this phenomenon remain unknown. It is interesting to note that previous studies, such as the one conducted by Bernardinelli [74], indicated that the proportion of insects reaching the adult stage can vary depending on the host plant, with preferences for species like *Quercus robur* L., *Q*. *pubescens*, *Q. petraea*, *Quercus cerris* L. (Fagaceae), *Rubus ulmifolius* Schott, and *Rubus idaeus* L. (Rosaceae).

These findings suggest that fluctuating climates may play a crucial role in the dynamics of OLB populations and could be an important factor in explaining the observed variation. Thus, further investigations are necessary to gain a deeper understanding of how the environment and climatic conditions affect this insect and contribute to the development of more effective management and conservation strategies.

### 4.4. Analysis of Differences in Egg Laying Trends among Generations

Our analysis of statistical parameters revealed that, from one generation to the next, females tended to lay a greater number of eggs per cluster. However, there was also a tendency to concentrate those eggs in a smaller number of clusters. The OLB exhibited variation in the number of eggs laid, with a range between one and nine clusters per female and between 1 and 125 eggs per cluster, depending on the generation. In contrast, Bernardinelli & Zandiagiacomo [22] indicated that the eggs of these insects were arranged in variable clusters, with numbers ranging from 15 to 100. This difference could be explained by the fact that, in this study, we tracked isolated pairs of insects and each generation separately, allowing for the observation of different behavioural responses to the environment and resource availability. These notable differences in the reproductive strategies of the OLB suggest that adaptation to environmental conditions, including access to food resources, plays a crucial role in shaping these behaviours.

In general, fecundity can be significantly influenced by the nutrition, quality, and quantity of food consumed by an insect both during its larval life and in adulthood [75]. The quality of host plant components, such as carbon, nitrogen, and defensive metabolites, directly affects the potential and fecundity of herbivorous insects [76]. Additionally, Ju et al. [68] found that temperature can have a significant impact on average fecundity in *C. ciliata*. Given the similarities between this species and the OLB, it is plausible that temperature could also influence the fecundity of the OLB.

These results lead us toward a deeper understanding of the complexity of adaptation in invasive species like the OLB to their new environments and to changes in resource availability. They also underscore the importance of continuing research to thoroughly examine how nutritional factors and the environment affect the reproductive behaviour of these insects, thus contributing to the development of more effective strategies for managing invasive insect populations

### 4.5. Practical Implications and Future Research Directions

Our results regarding the life cycle and degree day prediction model provide a comprehensive perspective on the evolution of this species. This information is essential for forest managers dealing with integrated pest management. The degree day prediction model offers a more precise method for anticipating critical moments in the development of this pest than alternative models, such as the calendar method. In general, it provides valuable information to crop managers regarding when a particular generation of pests reaches critical stages of development, allowing them to plan the timing of pesticide applications more efficiently [77]. Moreover, our prior study [78] highlighted challenges in chemically controlling the OLB due to its unique biology. These insights have the potential to improve chemical control efficacy by enabling more precise and efficient pesticide applications. Developing a life table of the insect population in a natural environment for the climatic conditions in Romania serves as a foundation for the development of a pest forecasting method. This life table not only enriches our knowledge of the species but also provides a solid basis for estimating how it can impact the environment [49,65].

From a pest management perspective, it is extremely valuable to identify when and under what circumstances a pest population experiences significant mortality, as this is the time when it becomes more susceptible to control measures [49,79]. Understanding critical moments in the life cycle of the OLB can lead to a reduction in the excessive use of pesticides. By focusing control treatments during periods when the OLB population is most vulnerable, the amount of pesticide required to keep the pest in check can be reduced. This not only reduces costs but also minimizes the negative effect of pesticides on the surrounding environment. This will help promote more sustainable forest management and protect fragile ecosystems.

Considering the invasive nature of the OLB, with a confirmed impact both in Romania and other European states, and the significant level of damage it can cause, continued, in-depth research is essential, especially given increasingly evident climate change. Thus, we believe that future research could take the following directions:

- Continuing periodic observations of the insect’s biology to refine and improve our degree day–based prediction model;
- Gaining a deeper understanding of how climatic conditions affect the life cycle and population density of the OLB;
- Developing advanced forecasting methods based on newly acquired knowledge;
- Assessing the potential risk of mass infestation in various ecosystems and geographic areas.

Acting on these research directions would help to contribute to a more comprehensive understanding of the interactions between the OLB and the environment and the development of more effective strategies for managing and mitigating the impact of the species on ecosystems and human activities. Additionally, such research could help adapt control measures to anticipated climate changes and support efforts to conserve forest ecosystems threatened by the invasion of the OLB.

## 5. Conclusions

In the climatic conditions of southeastern Romania, the OLB has three generations per year and overwinters in the adult stage, preferring sheltered locations. The first generation begins in the second half of May, followed by the second generation, which develops starting in July. Both the second and third generations conclude in November, coinciding with the withdrawal of adults for overwintering.

Our developed prediction model for the species, designed based on degree days, has the potential to significantly contribute to the development of a comprehensive population control program by forecasting key events in the biological cycle. It provides a valuable tool for the efficient management of this invasive species.

The life table developed for the insect population in the climatic conditions of Romania, based on indicators such as the number of adult pairs, proportion of females, number of eggs, and number of nymphs at various stages, along with the percentage of mortality at each developmental stage, explains the population dynamics of the insect across the three generations. This life table can serve as a valuable resource for the further development of more effective forecasting and management methods.

## Figures and Tables

**Table 1:** Installation dates of cloth bags.

Year	Generation	Date
2020	I	5 May 2020
	II	7 July 2020
	III	6 August 2020
2021	I	1 May 2021
	II	1 July 2021
	III	1 August 2021

**Table 2:** Developmental progression table for the oak lace bug population.

Index	The Generation of 2020			The Generation of 2021		
	G I	G II	G III	G I	G II	G III
*N*′	40	130	110	70	70	70
N	28	48	44	46	48	18
${lx}_{eggs}$	38.9 ± 5	58.4 ± 5.8	55 ± 4.2	25.9 ± 4.2	94.6 ± 10.3	52.1 ± 8.9
${dx}_{eggs}$	44.5%	34.3%	4.7%	6.2%	20.5%	34.3%
${lx}_{nymphs\;1}$	21.6 ± 2.8	38.4 ± 5.5	52.4 ± 4.4	24.3 ± 5.2	75.3 ± 8.7	34.2 ± 10.6
${dx}_{nymphs\;1}$	17.9%	11.6%	4.5%	6.3%	5.1%	9.1%
${lx}_{nymphs\;2}$	17.7 ± 2.6	30.7 ± 4.5	50.5 ± 4.6	22.7 ± 5.1	71.4 ± 8.7	31.1 ± 10.4
${dx}_{nymphs\;2}$	10.1%	9.6%	3.6%	5.4%	0.9%	10.4%
${lx}_{nymphs\;3}$	15.9 ± 2.6	29.2 ± 4.3	48.2 ± 4.6	21.5 ± 5	70.7 ± 8.6	27.9 ± 10.5
${dx}_{nymphs\;3}$	6.3%	4.6%	1.6%	1.2%	0.5%	8.4%
${lx}_{nymphs\;4}$	14.9 ± 2.7	28.2 ± 4.2	47.5 ± 4.7	21.3 ± 5	70.4 ± 8.6	25.6 ± 10.6
${dx}_{nymphs\;4}$	3.3%	3.6%	1.3%	0.4%	0.2%	2.2%
${lx}_{nymphs\;5}$	14.6 ± 2.7	27.4 ± 4.2	46.8 ± 4.8	21.2 ± 5	70.2 ± 8.6	25 ± 10.7
${dx}_{nymphs\;5}$	2%	3%	0.6%	0.4%	0.2%	32.4%
${lx}_{adults}$	14.1 ± 2.8	27.4 ± 4.2	46.5 ± 4.8	21.1 ± 5	70.1 ± 8.6	16.9 ± 11.5
${dx}_{female}$	0.4	0.5	0.5	0.5	0.5	0.5
GS	36%	47%	85%	81%	74%	32%

**Table 3:** Statistical metrics for egg laying trends across generations.

Characteristics	Statistical Parameters	Generation					
		I		II		III	
		2020	2021	2020	2021	2020	2021
Clusters/female	Mean	2.65	2.17	1.95	3.65	1.55	2.33
	Minimum	1	1	1	2	1	1
	Maximum	7	3	6	9	3	5
	Standard deviation	1.64	0.77	1.08	1.67	0.74	1.41
	Coefficient of variation (%)	62.68	35.77	56.09	45.67	48.07	60.61
Number of eggs/cluster	Mean	11.5	11.9	24.62	26.79	31.84	22.90
	Minimum	1	2	4	2	6	7
	Maximum	46	51	72	125	84	64
	Standard deviation	10.74	10.80	15.27	23.11	21.50	15.34
	Coefficient of variation (%)	94.10	90.71	62.02	86.27	67.23	66.97

## Data Availability

No new data were created or analysed in this study. Data are contained within the article.

## Appendix A

**Table A1:** Methodology for constructing the life table of the oak lace bug.

Index	Description	Method of Determination	Formula
*N*′	The total number of pairs established and introduced into the experiment.	Directly counting the actual number of the pairs established and introduced into the experiment.	N/A (directly counted).
*N*	The number of pairs that successfully completed the development cycle of the respective generation.	Directly counting the actual number of pairs that successfully completed the development cycle.	N/A (directly counted).
${lx}_{eggs}$	The average number of eggs per pair.	Directly counting the actual number of eggs produced by each pair at the end of oviposition and calculating the average for a pair.	N/A (directly counted).
${dx}_{eggs}$	The mortality and infertility of the eggs.	Determining the percentage of eggs that were non-viable due to natural enemies or other unknown biological causes and did not result in the development of first-instar nymphs.	${dx}_{eggs}=\left(\frac{{lx}_{nymphs\;1}}{{lx}_{eggs}}\right)\times 100$
${lx}_{nymphs\;1}$	The average number of first-instar nymphs.	Directly counting the number of nymphs resulting from the total number of eggs for each pair that survived until the end of the first stage of development and calculating the average for a pair.	N/A (directly counted).
${dx}_{nymphs\;1}$	The mortality of first-instar nymphs.	Determining the percentage of first-instar nymphs that did not survive until the end of the first instar due to natural enemies or other unknown biological causes.	${dx}_{nymphs\;1}=\left(\frac{{lx}_{nymphs\;2}}{{lx}_{nymphs1}}\right)\times 100$
${lx}_{nymhs\;2}$	The average number of second-instar nymphs.	Directly counting the number of nymphs resulting from the first-instar nymphs that survived until the end of the second instar and calculating the average for a pair.	N/A (directly counted).
${dx}_{nymphs\;2}$	The mortality of second-instar nymphs.	Determining the percentage of nymphs derived from first-instar nymphs that did not survive until the end of the second instar due to natural enemies or other unknown biological causes.	${dx}_{nymphs\;2}=\left(\frac{{lx}_{nymphs\;3}}{{lx}_{nymphs\;2}}\right)\times 100$
${lx}_{nymphs\;3}$	The average number of third-instar nymphs.	Directly counting the number of nymphs resulting from second-instar nymphs that survived until the end of the third instar and calculating the average for a pair.	N/A (directly counted).
${dx}_{nymhs\;3}$	The mortality of third-instar nymphs.	Calculating the percentage of nymphs originating from second-instar nymphs that did not survive until the end of the third instar due to natural enemies or other unknown biological causes.	${dx\;}_{nymphs\;3}=\left(\frac{{lx}_{nymphs\;4}}{{lx}_{nymphs\;3}}\right)\times 100$
${lx}_{nymphs\;4}$	The average number of fourth-instar nymphs.	Directly counting the number of nymphs resulting from third-instar nymphs that survived until the end of the fourth instar and calculating the average for a pair.	N/A (directly counted).
${dx}_{nymphs\;4}$	The mortality of fourth-instar nymphs.	Calculating the percentage of nymphs originating from third-instar nymphs that did not survive until the end of the fourth instar due to natural enemies or other unknown biological causes.	${dx}_{nymphs\;4}=\left(\frac{{lx}_{nymphs\;5}}{{lx}_{nymphs\;4}}\right)\times 100$
${lx}_{nymphs\;5}$	The average number of fifth-instar nymphs.	Directly counting the number of nymphs resulting from fourth-instar nymphs that survived until the end of the fifth instar and calculating the average for a pair.	N/A (directly counted).
${dx}_{nymphs\;5}$	The mortality of fifth-instar nymphs.	Determining the percentage of nymphs derived from fourth-instar nymphs that did not survive until the end of the fifth instar due to natural enemies or other unknown biological causes.	${dx}_{nymphs\;V5}=\left(\frac{{lx}_{adults}}{{lx}_{nymphs\;V5}}\right)\times 100$
${lx}_{adults}$	The average number of adults.	Directly counting the number of adults resulting from fifth-instar nymphs and calculating the average for a pair.	
${lx}_{female}$	The sex ratio.	The ratio of the number of females to the number of adults	${dx}_{female}=\left(\frac{{lx}_{female}}{{lx}_{adults}}\right)\times 100$
GS	The survival of the generation.	the population trend index. Without the effects of fertility and mortality of individuals after reaching adulthood.	$GS=\frac{{lx}_{adults}}{{lx}_{eggs}}\times 100$
